# Association between admission criteria and body composition among young children with moderate acute malnutrition, a cross-sectional study from Burkina Faso

**DOI:** 10.1038/s41598-020-69987-9

**Published:** 2020-08-06

**Authors:** Christian Fabiansen, Bernardette Cichon, Charles W. Yaméogo, Ann-Sophie Iuel-Brockdorf, Kevin P. Q. Phelan, Jonathan C. Wells, Christian Ritz, Suzanne Filteau, André Briend, Vibeke B. Christensen, Per Ashorn, Kim F. Michaelsen, Susan Shepherd, Henrik Friis

**Affiliations:** 1 Department of Nutrition, Exercise and Sports, University of Copenhagen, Rolighedsvej 25, 1958 Frederiksberg, Denmark; 2 Médecins Sans Frontières-Denmark, Dronningensgade 68, 3, 1420 Copenhagen, Denmark; 3 Département Biomédical et Santé Publique, Institut de Recherche en Sciences de la Santé, Ouagadougou 03, BP 7047 Bobo-Dioulasso, Burkina Faso; 4 ALIMA, Route de l’Aéroport, Rue NG 96, BP 15530 Dakar, Sénégal; 5 Childhood Nutrition Research Centre, UCL Great Ormond Street Institute of Child Health, 30 Guilford Street, London, WC1N 1EH UK; 6 London School of Hygiene and Tropical Medicine, Faculty of Epidemiology and Population Health, Keppel Street, London, WC1E 7HT UK; 7 Center for Child Health Research, Tampere University, Faculty of Medicine and Health Technology and Tampere University Hospital, Lääkärinkatu 1, 33014 Tampere, Finland; 8 Department of Paediatrics and Adolenscent Medicine, Blegdamsvej 9, 2100 RighospitaletCopenhagen, Denmark

**Keywords:** Nutrition, Paediatric research

## Abstract

Children with moderate acute malnutrition (MAM) are treated based on low weight-for-length z-score (WLZ), low mid-upper arm circumference (MUAC) or both. This study aimed to assess associations of admission criteria and body composition (BC), to improve treatment of MAM. We undertook a cross-sectional study among 6–23 months old Burkinabe children with MAM. Fat-free (FFM) and fat mass (FM) were determined by deuterium dilution and expressed as FFM (FFMI) and FM index (FMI). Of 1,489 children, 439 (29.5%) were recruited by low MUAC only (MUAC-O), 734 (49.3%) by low WLZ and low MUAC (WLZ-MUAC) and 316 (21.2%) by low WLZ only (WLZ-O). Thus, 1,173 (78.8%) were recruited by low MUAC, with or without low WLZ (ALL-MUAC). After adjustments, WLZ-O had 89 g (95% confidence interval (CI) 5; 172) lower FFM compared to MUAC-O. Similarly, WLZ-O had 0.89 kg/m^2^ (95% CI 0.77; 1.01) lower FFMI compared to MUAC-O, whereas there was no difference for FMI. However, boys included by WLZ-O compared to MUAC-O had 0.21 kg/m^2^ (95% CI 0.05; 0.38) higher FMI. In contrast, girls included by WLZ-O had 0.17 (95% CI 0.01; 0.33) kg/m^2^ lower FMI compared to MUAC-O (interaction, p = 0.002). We found that different criteria for admission into MAM treatment programmes select children with differences in BC, especially FFMI.

**Trial registration**: ISRCTN42569496.

## Introduction

Childhood malnutrition is associated with almost half the global mortality in children under 5 years of age^1^. Although both the prevalence and incidence of moderate acute malnutrition (MAM) is unknown, moderate wasting alone affects 33 million children at any time^2^ and is associated with a threefold increased risk of death^3^.

MAM is currently defined as weight-for-length z-score (WLZ) between − 3 and − 2 (i.e. moderate wasting), and/or mid-upper arm circumference (MUAC) between 115 and 125 mm^4^. These criteria based on standard anthropometry are used both for inclusion in MAM programs and eligibility for trials assessing the effect of different supplements^5–11^.

MUAC and WLZ have been used independently to determine eligibility for nutritional therapy and identify overlapping but not identical groups of children^12^. MUAC is increasingly used as the sole anthropometric admission criterion for nutrition programs, taking advantage of its simplicity of use^13^.

Children detected by universal MUAC thresholds are on average younger, shorter and more likely to be female while those identified by gender-specific WLZ are on average older, longer and male^14^. However, little is known about how these anthropometric parameters relate to body composition, which is a more accurate way to evaluate nutritional status and likely to be key in short term response to treatment and for predicting longer term risks of non-communicable diseases^15^. Moreover, wasting and stunting have been shown to be interrelated conditions but so far body composition in stunted children with MAM has only been described in children admitted with MUAC as single criterion^16^. Here we present data on stunted children with MAM irrespective of their admission criteria. Indices of body composition, i.e. fat and fat-free mass^17^, can be determined under field conditions using the deuterium dilution technique^18^.

This was a cross-sectional study using trial cohort baseline data on body composition^18^. Our objective was to assess the association of both types of anthropometric admission criteria with body composition and to describe body composition in stunted children with MAM.

## Methods

This study was part of the Treatfood trial, a randomized trial with a 2 × 2 × 3 factorial design, investigating the effectiveness of 500 kcal/day supplement either as corn-soy blend (CSB) porridge or ready-to-use lipid-based nutrient supplements (LNS) for the treatment of MAM^18^. As previously described the LNS supplements provided almost three times more energy as fat than the CSB supplements (~ 57% vs ~ 21%). Assignment to one of the 12 supplements (6 CSB and 6 LNS) followed randomisation stratified by site.

### Participants

Data were collected in the Province du Passoré in the Northern region of Burkina Faso at five research sites located at different governmental health centers (Gomponsom, Latoden, Bagaré, Bokin and Samba) and staffed by the non-governmental organization Alliance for International Medical Action (ALIMA, Dakar, Senegal).

Children were screened in villages either by community health workers using MUAC tapes or by designated screening teams with the use of both MUAC and WLZ. Moreover, children could be referred from a health centre or could present at site on caretaker´s initiative. At the sites, the final assessment of eligibility for inclusion was performed.

Children aged 6–23 months with MAM (defined as MUAC between 115 and 125 and/or a WLZ between − 3 and − 2), resident in the catchment area and whose parent and/or legal guardian gave informed consent, were included. Children were not included if treated for severe acute malnutrition (SAM) or hospitalized within the past two months, if already in a nutritional program or if they presented medical complications requiring hospitalization. Likewise, children with a severe disability limiting the possibility of investigations and children with suspected allergy to milk, peanuts, corn or soy were not included.

### Procedures and study visits

We previously described clinic visits, standard anthropometric measurements and age determination^18^. In the present paper we report on indices of body composition and skinfolds assessed at baseline. Total body water (TBW) was assessed using the deuterium dilution technique for assessment of fat-free mass (FFM) and fat mass (FM). The method involved giving an oral dose of 5 g deuterium oxide (D_2_O) (99.8%, Cambridge Isotope Laboratories Inc., Andover, USA). The isotope was diluted in 5 g of bottled water (LAFI, Burkina Faso), with the dosing bottle weighed with 0.01 g precision (Adam equipment: model CQT 202, United Kingdom) before and after administration of the dose. Pre-dose saliva samples were obtained to assess background isotope levels in body fluids, and post-dose saliva samples were collected after a three-hour equilibration period as established during the pilot study^19^. For each assessment, deuterium enrichment was measured in duplicate in the pre- and post-dose saliva samples and in a diluted sample of the dose, using Fourier Transform Infrared Spectrometry (FTIR,Agilent Technologies, CA, USA)^20^ at St. John's Research Centre, Bangalore, India. Saliva samples required at least 60 µl saliva for analysis. Deuterium dilution space was calculated as described previously ^21^, and converted to total body water (TBW) using a factor of 1.044 to adjust for proton exchange^22^. FFM was calculated as TBW/hydration, using age- and sex-specific hydration coefficients^23^. FM was calculated as weight minus FFM. Data were cleaned for typographical errors and implausible TBW values, based on the association of TBW with length and cut-offs for FM of < − 0.1 and > 2.4 kg.

Anthropometric measurements were undertaken by trained staff, after standardization sessions. Skinfold thickness was measured by a Harpenden caliper. Weight was measured to the nearest 100 g using electronic scales (Seca model 8811021659) with double weighing function. Length was measured with a wooden length board to the nearest 1 mm. WLZ was determined at sites using WHO sex-specific field tables, and this value was used for recruitment. MUAC was measured to the nearest 1 mm, at the midpoint between the olecranon and the acromion process using a standard measuring tape. For all anthropometric measures, the mean of the duplicate measurements was taken for analysis.

In later analyses, WLZ, length-for-age z-score (LAZ) and weight-for-age z-score were calculated using the package “zscore06” in Stata 12 (College Station, Texas, USA). Skinfold-for-age z-scores were calculated using WHO's Anthro Plus software (version 3.2.2, 2011, World Health Organization, Geneva, Switzerland). All z-scores were calculated using the 2006 WHO child growth standards^24^.

At enrolment, a research nurse collected data on demographic characteristics, vaccination status, and 2-week retrospective morbidity and medical treatments using a structured questionnaire in the local language. Data on birthweight was acquired if children presented with a health card providing the information, accordingly, measurement of birthweight was not standardized within the cohort. All children received vitamin A supplementation (100,000 International Units (IU) if 4–8 kg; 200,000 IU if > 8 kg) if they had not received any supplements in the previous 6 months, and albendazole (200 mg if 4–8 kg; 400 mg if > 8 kg) and vaccinations were administered according to the national schedule at the health centres.

### Outcomes

Body composition was evaluated using a two-component model, differentiating FFM i.e., muscle, organs, and bone, and FM. As age differs considerably between admission groups, indices of body composition adjusted for length, which is closely correlated with age, were chosen as main outcome. The fat-free mass index (FFMI) and fat mass index (FMI) were obtained by dividing FFM and FM by length squared, giving indices expressed in kg/m^2^, similar to body mass index (BMI).

We primarily compared body composition between the three admission groups used in the main trial those admitted based on low MUAC only (MUAC-O), both low WLZ and low MUAC (WLZ-MUAC) and low WLZ only (WLZ-O). For reasons of simplicity, to improve coverage and to facilitate screening by caretakers, malnutrition programs increasingly use MUAC only (admission by low MUAC as single criterion)^25^. We therefore also simulated the effect of this strategy and compared all children that would have been included in a MUAC-only programme (ALL-MUAC) vs children only qualifying by WLZ only: ALL-MUAC (MUAC-O + WLZ-MUAC) vs WLZ-O. These groups will be referred to as operational groups.

### Statistical analysis

Data were double entered in Epidata 3.1 (Epidata Association, Odense, Denmark) and double entry checks were performed daily. All statistical analyses were carried out using the statistical software package Stata version 12 (StataCorp, College station, Texas, USA).

Differences between MUAC-O, WLZ-MUAC, and WLZ-O were evaluated by a chi-square test or a one-way ANOVA. P-values from post-hoc pairwise comparisons were Bonferroni adjusted.

Linear mixed models were used to assess associations of admission criteria with indices of body composition. We considered both unadjusted models and models adjusted for age, sex, month of inclusion and site (random effects). Additionally, for the adjusted models, interactions with sex were evaluated and, in case of a significant interaction, stratified analyses were carried out. Model checking was based on residuals and normal probability plots. A significance level of 0.05 was applied.

### Role of the funding source

The funder of the study had no role in study design, data collection, data analysis, data interpretation, or writing of the report. The corresponding author had full access to all the data from the study and had final responsibility for the decision to submit for publication.

### Informed consent and ethics

Written informed consent was obtained from all caregivers (signature or fingerprint) prior to enrolment. The trial was approved by the Ethics Committee for Health Research in Burkina Faso (2012-8-059) and consultative approval was obtained from the Danish National Committee on Biomedical Research Ethics (1208204). Trial registration: ISRCTN42569496. All research was performed in accordance with relevant guidelines and regulations.

## Results

Of 1609 children in the trial, data on body composition were available on 1,489 (92.5%). Among these, the mean standard deviation (SD) age was 12.4 (4.8) months, 821 (55.1%) were females and 988 (66.4%) were included in the dry season. Most children 1,404 (94.4%) were still breastfed. The mean (SD) MUAC was 123 (4) mm and mean (SD) length was 70.5 (5.3) cm. As previously reported^18^, the mean (SD) weight of 6.92 (0.92) kg comprised 5.79 (0.91) kg FFM and 1.13 (0.39) kg FM. The mean (SD) FFMI was 11.62 (0.87) kg/m^2^ and FMI was 2.30 (0.78) kg/m^2^. The mean (SD) WLZ was − 2.21 (0.51). The mean (SD) weight for age z-score (WAZ) was − 2.51 (0.65) and mean (SD) LAZ was − 1.68 (1.12), with 552 (37.1%) being stunted (LAZ < − 2) and 149 (10%) being severely stunted (LAZ < − 3). Mean (SD) triceps skinfold-for-age z-score was − 1.43 (0.85) and subscapular skinfold-for-age z-score − 1.59 (0.94). The pattern of variability in skinfolds across the three groups was consistent with the equivalent pattern for fat mass, with the WLZ-MUAC group showing lowest values in both cases.

**Figure 1:**
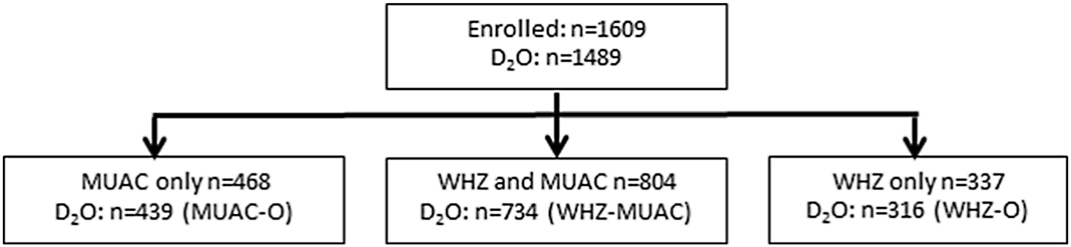
Participant flow chart. Adapted from Ref.^18^. Body composition assessment by deuterium dilution (D_2_O). Mid-upper arm circumference (MUAC). Weight-for-length z-score (WLZ). Children with D_2_O admitted by MUAC only (MUAC-O). Children with D_2_O admitted by WLZ and MUAC (WLZ-MUAC). Children with D_2_O admitted by WLZ only (WLZ-O).

Of the 1,489 children with body composition data, 439 (29.5%) were recruited by MUAC-O, 734 (49.3%), were recruited based on WLZ-MUAC and 316 (21.2%) by WLZ-O (Fig. 1). In unadjusted analysis those recruited by low MUAC (i.e. either MUAC-O or WLZ-MUAC) were more likely to be girls, and were younger, shorter and weighed less (Table 1).

**Table 1:** Characteristics of 1,489 children with moderate acute malnutrition by admission criteria.

	MUAC-O (n = 439)	WLZ-MUAC (n = 734)	WLZ-O (n = 316)	p-value^1^
**Background characteristics**				
Girls, % (n)	78.1 (343)^a^	52.6 (386)^b^	29.1 (92)^c^	**< 0.001**
Age, months	11.1 (4.6)^a^	12.6 (4.8)^b^	13.6 (4.8)^c^	**< 0.001**
Season at inclusion, % (n)				0.24
Dry season	64.7 (284)	65.7 (482)	70.3 (222)	
Rainy season	35.3 (155)	34.3 (252)	29.8 (94)	
Site, % (n)				**< 0.001**
0	19.6 (86)	24.0 (176)	23.7 (75)	
1	13.2 (58)^a^	18.0 (132)^a^	34.5 (109)^b^	
2	19.1 (84)	16.8 (123)	15.5 (49)	
3	28.0 (123)^a^	24.9 (183)^a^	10.4 (33)^b^	
4	20.1 (88)	16.4 (120)	15.8 (50)	
Breastfeeding, % (n), (n = 1,487)	95.0 (417)	94.0 (688)	94.6 (299)	0.76
Ill past 2 weeks, maternal recall % (n), (n = 1,480)	34.3 (150)	38.6 (281)	40.3 (127)	0.19
**Standard anthropometry**				
Weight (kg)	6.78 (0.97)^a^	6.81 (0.88)^a^	7.37 (0.81)^b^	**< 0.001**
Length (cm)	68.2 (5.2)^a^	70.7 (4.9)^b^	73.1 (4.8)^c^	**< 0.001**
Mid-upper arm circumference (mm)	121.6 (2.4)^a^	120.8 (2.6)^b^	128.5 (2.9)^c^	**< 0.001**
Weight-for-length z-score	− 1.59 (0.34)^a^	− 2.50 (0.30)^b^	− 2.41 (0.28)^c^	**< 0.001**
Weight-for-age z-score	− 2.22 (0.61)^a^	− 2.72 (0.63)^b^	− 2.44 (0.57)^c^	**< 0.001**
Length-for-age z-score	− 1.83 (1.02)^a^	− 1.72 (1.16)^a^	− 1.39 (1.10)^b^	**< 0.001**
< − 2% (n), (n = 552)	41.9 (184)^a^	38.2 (280)^a^	27.9 (88)^b^	**< 0.001**
< − 3% (n), (n = 149)	10.5 (46)^a,b^	11.6 (85)^a^	5.7 (18)^b^	**0.013**^2^
**Body composition**				
Fat-free mass (kg)	5.62 (0.97)^a^	5.75 (0.88)^a^	6.11 (0.84)^b^	**< 0.001**^3^
Fat-free mass index (kg/m^2^)	12.03 (0.89)^b^	11.46 (0.80)^a^	11.41 (0.80)^a^	**< 0.001**
Fat mass (kg)	1.16 (0.38)^a^	1.07 (0.37)^b^	1.26 (0.41)^c^	**< 0.001**
Fat mass index (kg/m^2^)	2.51 (0.83)^a^	2.14 (0.72)^b^	2.37 (0.75)^c^	**< 0.001**
Triceps skinfold-for-age z-score, (n = 1,487)	− 1.40 (0.82)^a^	− 1.61 (0.82)^b^	− 1.05 (0.84)^c^	**< 0.001**
Subscapular skinfold-for-age z-score (n = 1,488)	− 1.42 (0.91)^a^	− 1.79 (0.91)^b^	− 1.36 (0.93)^a^	**< 0.001**

Data are mean (± SD) unless otherwise indicated.

^1^Overall p-value for chi-square or F test from ANOVA, means inside table not sharing a superscript letter are different following Bonferonni correction. P-values below 0.05 are in bold.

^2^Height-for-age z-score, < − 3% comparison WLZ-O vs MUAC-O, p = 0.06.

^3^Fat-free mass MUAC-O vs WLZ-MUAC: p = 0.06.

In Table 2 we present differences in body composition by admission criteria with site as random effect in unadjusted and adjusted models. As seen, admission by WLZ-O was associated with 471 g (95% confidence interval (CI) 340; 602) greater FFM compared to admission by MUAC-O. However, after adjustment for age, sex and month of inclusion, admission by WLZ-O was associated with 89 g (95% CI 5; 172) lower FFM. Similarly, in adjusted analysis the WLZ-O had 0.89 kg/m^2^ (95% CI 0.77; 1.01) lower FFMI (i.e. FFM indexed by length) compared to the MUAC-O group, whereas there was no difference for FMI. Table 2 also presents differences in body composition between children admitted by WLZ-O compared to ALL-MUAC. As seen, those admitted with WLZ-O had 396 g (95% CI 283; 508) greater FFM in the unadjusted analysis, whereas there was no significant difference after adjustment (63 g, 95% CI − 8; 134). Conversely, admission by WLZ-O was associated with 0.37 kg/m^2^ (95% CI 0.27; 0.48) lower FFMI and 0.23 kg/m^2^ (95% CI 0.14; 0.32) greater FMI, compared to those admitted based on ALL-MUAC.

**Table 2:** Differences in body composition among 1,489 children with moderate acute malnutrition, according to admission citeria.

	Fat-free mass (g)		Fat-free mass index (kg/m^2^)		Fat mass (g)		Fat mass index (kg/m^2^)	
	Unadjusted	Adjusted	Unadjusted	Adjusted	Unadjusted	Adjusted	Unadjusted	Adjusted
**Admission criteria**^**1**^								
MUAC only (n = 439)	Ref	Ref	Ref	Ref	Ref	Ref	Ref	Ref
MUAC and WLZ (n = 734)	**118 (13;224)**	**− 209 (− 274; − 145)**	**− 0.58 (− 0.68; − 0.48)**	**− 0.71 (− 0.80; − 0.62)**	**− 84 (− 128; − 40)**	**− 86 (− 130; − 43)**	**− 0.35 (− 0.44; − 0.26)**	**− 0.26 (− 0.35; − 0.18)**
WLZ only (n = 316)	**471 (340;602)**	**− 89 (− 172; − 5)**	**− 0.64 (− 0.76; − 0.52)**	**− 0.89 (− 1.01; − 0.77)**	**119 (64;174)**	**114 (58;170)**	**− **0.10 (**− **0.21;0.01)	0.04 (**− **0.07;0.15)
**Operational admission criteria**^**2,3**^								
ALL-MUAC (n = 1,173)	Ref	Ref	Ref	Ref	Ref	Ref	Ref	Ref
WLZ only (n = 316)	**396 (283;508)**	63 (**− **8;134)	**− 0.27 (− 0.38; − 0.16)**	**− 0.37 (− 0.48; − 0.27)**	**173 (126;220)**	**177 (130;223)**	**0.12 (0.03;0.22)**	**0.23 (0.14;0.32)**
**Length-for-age Z category**								
≥ **− **2 (n = 937)	Ref	Ref	Ref	Ref	Ref	Ref	Ref	Ref
≥ **− **3.0 to **− **2.0 (n = 403)	**− **61 (**− **166;45)	**− 407 (− 461; − 353)**	**0.30 (0.20;0.40)**	**0.30 (0.21;0.40)**	**− 81 (− 125; − 37)**	**− 97 (− 140; − 55)**	**− **0.08 (**− **0.17;0.01)	0.02 (**− **0.06;0.11)
< **− **3.0 (n = 149)	**− 247 (− 404; − 91)**	**− 906 (− 986; − 825)**	**0.34 (0.19;0.48)**	**0.33 (0.18;0.47)**	**− 133 (− 199; − 68)**	**− 168 (− 231; − 105)**	**− **0.11 (**− **0.24;0.02)	0.07 (**− **0.06;0.20)

Data are estimated mean difference (95% CI) from linear mixed models with site as random effect in unadjusted and adjusted model. Adjustments include age, sex and month of inclusion. Estimates in bold are significant, p < 0.05.

^1^Analysis for interaction between admission criteria and sex showed interactions in model adjusted for age and months of admission and site (random effects): FFM, p = 0.002; FFMI, p = 0.051; FM, p = 0.017: FMI, p = 0.002.

^2^Operational admission criteria: ALL-MUAC (MUAC only + MUAC and WLZ) vs WLZ only.

^3^Analysis for interaction between operational admission criteria and sex showed significant interactions in model adjusted for age, months of admission and site (random effects) with p-values FFM, p = 0.007; FFMI, p = 0.43; FM, p = 0.002; FMI, p = 0.0001. Sex-stratified analyses of indices of body composition and all admission criteria are shown in Table 3.

After adjustments, moderate stunting (LAZ ≥ − 3.0 to − 2.0) and severe stunting (LAZ < − 3.0) were associated with 407 g (95% CI 353; 461) and 906 g (95% CI 825; 986) lower FFM, respectively, and 97 g (95% CI 55; 140) and 168 g (95% CI 105; 231) lower FM, respectively, compared to children without stunting (Table 2). In contrast, FFMI was 0.30 kg/m^2^ (95% CI 0.21; 0.40) greater among children with moderate stunting, and 0.33 kg/m^2^ (95% CI 0.18; 0.47) greater among children with severe stunting, compared to non-stunted children, whereas there were no differences in FMI.

Since the associations between admission criteria and the body composition outcomes shown in Table 2 largely depended on sex (see footnotes in Table 2 for p-values for all interactions), we also present the associations stratified by sex (Table 3). As seen, average FFMI for boys included by WLZ-O was 1.00 (95% CI 0.82; 1.18) kg/m^2^ lower than those included by MUAC-O, whereas average FFMI for girls included by WLZ-O was 0.73 (95% CI 0.56; 0.90) kg/m^2^ lower than for girls included by MUAC-O (interaction between admission criteria and sex for FFMI, p = 0.051). Interestingly, boys included by WLZ-O compared to those included by MUAC-O had 0.21 kg/m^2^ (95% CI 0.05; 0.38) higher FMI. In contrast, girls included by WLZ-O had 0.17 (95% CI 0.01; 0.33) kg/m^2^ lower FMI compared to MUAC-O (interaction between admission criteria and sex for FMI, p = 0.002). Similarly, compared to admission by ALL-MUAC, admission by WLZ-O was associated with 0.36 kg/m^2^ (95% CI 0.25; 0.48) higher FMI among boys, but not among girls (− 0.02, 95% CI − 0.17; 0.14) (interaction, p = 0.0001). There was no difference between boys and girls with respect to FFMI (− 0.40 vs − 0.32, interaction, p = 0.43).

**Table 3:** Sex-stratified differences in body composition by admission criteria among 1,489 children with moderate acute malnutrition.

	Fat-free mass (g)	Fat-free mass index (kg/m^2^)	Fat mass (g)	Fat mass index (kg/m^2^)
**Admission criteria boys (n = 668)**				
MUAC only (n = 96)	Ref	Ref	Ref	Ref
MUAC and WLZ (n = 348)	**− 276 (− 394; − 158)**	**− 0.75 (− 0.91; − 0.58)**	**− **65 (**− **143; 15)	**− 0.19 (− 0.34; − 0.03)**
WLZ only (n = 224)	**− 226 (− 352; − 99)**	**− 1.00 (− 1.18; − 0.82)**	**178 (94;263)**	**0.21 (0.05:0.38)**
**Admission criteria girls (n = 821)**				
MUAC only (n = 343)	Ref	Ref	Ref	Ref
MUAC and WLZ (n = 386)	**− 197 (− 274; − 120)**	**− 0.71 (− 0.82; − 0.60)**	**− 87 (− 138; − 35)**	**− 0.27 (− 0.37; − 0.17)**
WLZ only (n = 92)	76 (**− **48, 199)	**− 0.73 (− 0.90; − 0.56)**	26 (**− **56; 108)	**− 0.17 (− 0.33: − 0.01)**
**Operational admission criteria**^**1,2**^** boys (n = 668)**				
ALL-MUAC (n = 444)	Ref	Ref	Ref	Ref
WLZ only (n = 224)	**− **6 (**− **92; 81)	**− 0.40 (− 0.53; − 0.27)**	**230 (172; 288)**	**0.36 (0.25; 0.48)**
**Operational admission criteria girls (n = 821)**				
ALL-MUAC (n = 729)	Ref	Ref	Ref	Ref
WLZ only (n = 92)	**191 (74; 308)**	**− 0.32 (− 0.49; − 0.14)**	76 (**− **1;154)	**− **0.02 (**− **0.17;0.14)

Data are estimated mean difference (95% CI) from linear mixed models. Sex stratified analysis were adjusted for age and month of inclusion and site (random effect). Estimates in bold are significant, p < 0.05.

^1^Operational admission criteria: ALL-MUAC: ((MUAC only) + (MUAC and WLZ) vs (WLZ only)). As also shown in Table 2, analysis for interaction between admission criteria and sex showed interactions in model adjusted for age and months of admission and site (random effect).: FFM, p = 0.002; FFMI, p = 0.051; FM, p = 0.017: FMI, p = 0.002. Operational admission criteria: ALL-MUAC: ((MUAC only) + (MUAC and WLZ) vs (WLZ only)).

^2^Analysis for interaction between operational admission criteria and sex showed significant interactions in model adjusted for age, months of admission and site (random effects) with p-values FFM, p = 0.007; FFMI, p = 0.43; FM, p = 0.002; FMI, p = 0.0001.

The associations between admission criteria and the body composition outcomes shown in Table 2 also differed according to the presence or absence of stunting at admission. We therefore also assessed the associations after stratification by stunting (Table 4). As seen, the deficits in FFM and FFMI among children included by WLZ-O compared to MUAC-O were greater among stunted compared to non-stunted children, although only marginally significant for the FFMI (interactions non-stunted children vs stunted children and admission criteria with respect to FFM, p = 0.025, and FFMI, p = 0.078). Furthermore, the higher FM and FMI in children included by WLZ-O compared to MUAC-O was mainly seen in stunted children (interactions non-stunted children vs stunted children and admission criteria with respect to FM, p = 0.006, and FMI, p = 0.006). Likewise, compared to admission by ALL-MUAC, admission by WLZ-O was associated with 0.49 kg/m^2^ (95% CI 0.31; 0.68) lower FFMI and 0.45 kg/m^2^ (95% CI 0.29; 0.61) higher FMI among stunted children, compared to 0.24 (95% CI 0.12; 0.36) lower FFMI and 0.16 kg/m^2^ (95% CI 0.05; 0.27) higher FMI among non-stunted children (interaction, p < 0.05).

**Table 4:** Stunting-stratified differences in body composition by admission criteria among 1,489 children with moderate acute malnutrition.

	Fat-free mass (g)	Fat-free mass index (kg/m^2^)	Fat mass (g)	Fat mass index (kg/m^2^)
**Not-stunted (n = 937)**^**1**^				
MUAC only (n = 255)	Ref	Ref	Ref	Ref
MUAC and WLZ (n = 454)	**− 252 (− 324; − 182)**	**− 0.67 (− 0.79; − 0.56)**	**− 112 (− 166; − 58)**	**− 0.29 (− 0.39; − 0.18)**
WLZ only (n = 228)	**− 200 (− 288; − 112)**	**− 0.75 (− 0.89; − 0.61)**	29 (**− **37; 96)	0.05 (**− **0.18; 0.08)
**Stunted (n = 552)**^**2**^				
MUAC only (n = 184)	Ref	Ref	Ref	Ref
MUAC and WLZ (n = 280)	**− 326 (− 413; − 238)**	**− 0.71 (− 0.85; − 0.57)**	**− 83 (− 150; − 17)**	**− **0.21 (**− **0.35;** − **0.08)
WLZ only (n = 88)	**− 398 (− 520, − 276)**	**− 1.00 (− 1.19; − 0.81)**	**199 (106;291)**	**0.29 (0.11:0.48)**
**Operational admission criteria** **Not-stunted (n = 937)**				
ALL-MUAC (n = 709)	Ref	Ref	Ref	Ref
WLZ only (n = 228)	**− **4 (**− **79; 70)	**− 0.24 (− 0.36; − 0.12)**	**112 (57; 168)**	**0.16 (0.05; 0.27)**
**Operational admission criteria**^**3**^ **Stunted (n = 552)**				
ALL-MUAC (n = 464)	Ref	Ref	Ref	Ref
WLZ only (n = 88)	**− 167 (− 278; − 56)**	**− 0.49 (− 0.68; − 0.31)**	**260 (178; 342)**	**0.45 (0.29; 0.61)**

Data are estimated mean difference (95% CI) from linear mixed models. Analysis stratified for stunting were adjusted for age month of inclusion, sex and site (random effect). Estimates in bold are significant, p < 0.05.

^1^Not stunted (length for z score ≥ **− **2).

^2^Stunted (length for z score < **− **2). In the adjusted model, interactions were identified between non-stunted children vs stunted children and admission criteria with respect to FFM, p = 0.025; FM, p = 0.006; FMI, p = 0.006, whereas it was marginally significant for FFMI (p = 0.078).

^3^Operational admission criteria: ALL-MUAC: ((MUAC only) + (MUAC and WLZ) vs (WLZ only)). Analysis for interaction between operational admission criteria and stunting showed significant interactions in model adjusted for age, months of inclusion, sex and site (random effects) with p-values FFM, p = 0.014 ; FFMI, p = 0.021 , FM, p = 0.003 ; FMI, p = 0.003.

## Discussion

Admission to a MAM intervention programme is based on falling below specific cutoffs of standard anthropometric measurements of MUAC and WLZ. It is well established that MUAC and WLZ identify overlapping but not identical groups of children. There may be other longer term health risks associated with early childhood malnutrition that could be elucidated by investigating the relative changes in lean and fat tissues during nutrition supplementation. To shed new light on these issues, we investigated how the different approaches used to recruit children result in differing body composition at onset of treatment.

This was a cross-sectional study using baseline data from a trial, which has been reported previously without consideration of variability by admission criteria^18^. To conduct these comparisons, we adjusted for age, as the groups admitted by different criteria varied in their average age. For similar reasons, we also adjusted for the calendar month of measurement, and for the site where the child was measured. In addition, we also conducted analyses first without (i.e. FFM and FM), and then with, adjustment for length (i.e. the indices of FFMI and FMI).

Compared to those recruited by MUAC only, those recruited by WLZ only had lower FFMI. If these analyses were stratified by sex, then the deficit in FFMI was slightly stronger in males than females. In addition, among those recruited by WLZ only compared to MUAC only, boys had greater whereas girls had lower FMI. Our overall results show some consistency with a previous study of healthy infants in Ethiopia, where WLZ was likewise shown to be a stronger correlate than MUAC of variability in lean mass^26^. In terms of absolute tissue masses, the results indicate that malnourished males show greater penalties in FFM compared to females. In good conditions, males have greater FFM than females, but this means that they require higher energy requirements to support it, and may therefore lose relatively more FFM during malnutrition. The sex difference was reduced when the outcome was FFMI.

Overall, the deficits of WLZ are greater for FFM and FFMI, than for FM and FMI. This makes sense from a biological perspective: lean tissue is a high-cost high-reward tissue, whose fitness benefits lie in part in the future, but whose maintenance costs manifest during early childhood. During malnutrition, lean mass is therefore sacrificed and although fat is also used to buffer energy shortfalls, maintaining fat becomes increasingly important for survival. Some studies have shown that low levels of leptin a hormone secreted by fat and important for immune function, predict mortality in malnourished children^27,28^.

By enabling comparison to international reference data, the data on skinfolds confirm the malnourished condition of this population, with mean values being below -1 z-scores for all three groups, and -1.8 z-scores for subscapular in the WLZ-MUAC group. In two of the three groups (those incorporating WLZ in the criteria for admission), the subscapular z-score was lower than the triceps z-score. Central body fat is more closely associated than peripheral fat with immune function^29,30^, moreover central body fat may also supply energy for immune function which is costly^31^. Consistent with that, previous work in adults has shown that higher pathogen burdens are associated with lower subscapular skinfold, suggesting greater use of energy from this fat depot to fund immune function^32^. Therefore, these data may indicate a greater contribution of the costs of infection to the poorer nutritional status of the WLZ-MUAC group,however, this requires direct confirmation.

If the reference group was extended to include all children for whom low MUAC defined admission, the deficit in FFMI was smaller, and did not interact with sex, and accompanied by a higher FMI, although the latter was only seen in boys.

Wasting and stunting have been shown to be interrelated conditions, both associated with increased mortality and affecting the same population and often the same child^33,34^. The effect on mortality is especially strong when both are present^35^. In our data, children identified by All MUAC had higher rates of stunting and nearly double the rates of severe stunting compared to those identified by WLZ only. This is consistent with a previous report in Ethiopian infants, which found that MUAC was more sensitive than WLZ to variability in length/height^26^. Overall, stunted children had higher FFMI, more so if they were severely stunted, indicating that the deficits in length were greater than those in absolute FFM. There was no difference in FMI between stunted and non-stunted children.

When we stratified the comparison of body composition between the admission groups by stunting status, we found that the average deficits in FFMI and surplus FMI of the WLZ group were greater in the stunted children. Conversely, there was more variability in FMI across the admission groups, with stunted WLZ children having greater FMI than MUAC only children, whereas this difference was not evident in non-stunted children. The results for the comparison of MUAC and WLZ children with MUAC only children showed the same pattern, but the magnitudes of effect tended to be lower. Using all-MUAC children as the reference, the differences of the WLZ children were again greater in stunted versus non-stunted children. These findings are consistent with those of other studies, which have reported deficits in FFM in stunted children, sometimes accompanied by a relative preservation of fat mass^36^.

On this basis, if WLZ is not used to recruit children, there will be a tendency to miss those with lower FFMI. This will be the case for both boys and girls, but among boys, in contrast to girls, the lower FFMI will be accompanied by a higher FMI.

We do not believe that these results currently have any implications for practice. First, the measurement of weight-for-length requires costly and bulky equipment and the ability to read weight-for-length tables. While this is possible to do at health centres it is not practical for detection of malnutrition at a large scale involving community health volunteers or family or family members. MUAC bracelets are low cost and can be used with minimal training by both health workers and family members at large scale, and due to colour coding does not require the ability to read or write. The use of MUAC at the community level can therefore dramatically increase coverage of early detection of children with acute malnutrition at large scale and improve efficiencies in which programs reach vulnerable children.

Second, as yet, we know little about the relative importance of FFMI versus FMI for immediate survival in moderately malnourished young children. Fat provides energy for a range of body functions including the immune system, whereas muscle provides proteins also needed for the inflammation response and immune system^37^. In the long term, low FFMI is associated with increased risk of non-communicable diseases^38^. In the entire sample, the average ratio of fat to fat free mass is ~ 1 to 5, regardless of the admission criteria used. For comparison, the equivalent ratio is 1–3 in healthy European 1–2 year olds^39^. Such a difference is unsurprising given the fact that fat is used as fuel for metabolism of vital organs such as the liver and heart during malnutrition^40,41^. There is ample evidence that stunting in early childhood is associated with long-term health and educational impacts^42,43^. However, the relative value of lean and fat mass, or the ratio between the two, in malnourished children is less certain, as is what constitutes the optimum accretion of either during recovery from malnutrition^17^. In non-infected patients, physiology suggests that fat is the main determinant of survival during starvation^41^. In infected patients, lean tissue may be more important^33^, but recently studies have shown that lower leptin (a marker of fat) in hospitalized children with very low anthropometric measures was a predictor of mortality^27,28^. The relative importance of fat-free and fat mass may also vary according to season^44^. These are important questions for future studies to address.

The strengths of the study include a large group of children and use of the deuterium dilution technique which provides objective data on the relative proportions of fat-free and fat mass in body weight. We also looked at several significant interactions including sex and stunting status. Although the aim of our study (i.e. to describe body composition among MAM children by admission criteria) obviously requires a cross-sectional study design, we cannot exclude the possibility of selection bias.

In conclusion we found that different criteria for admission into treatment programmes in moderate malnutrition select children with subtle but significant differences in body composition, especially FFMI. However, to fully understand the significance of these differences, we need more information on the importance of fat and fat-free tissue in malnourished children, and to assess if baseline differences in fat and fat-free mass modify the response to treatment. Moreover, there may be other outcomes, aside from body composition, that show larger contrasts between children admitted by MUAC or WLZ criteria. Therefore, the issue merits further investigation.
