# Publisher Correction: Association between admission criteria and body composition among young children with moderate acute malnutrition, a cross-sectional study from Burkina Faso

**DOI:** 10.1038/s41598-020-73323-6

**Published:** 2020-10-06

**Authors:** Christian Fabiansen, Bernardette Cichon, Charles W. Yaméogo, Ann-Sophie Iuel-Brockdorf, Kevin P. Q. Phelan, Jonathan C. Wells, Christian Ritz, Suzanne Filteau, André Briend, Vibeke B. Christensen, Per Ashorn, Kim F. Michaelsen, Susan Shepherd, Henrik Friis

**Affiliations:** 1 Department of Nutrition, Exercise and Sports, University of Copenhagen, Rolighedsvej 25, 1958 Frederiksberg, Denmark; 2 Médecins Sans Frontières-Denmark, Dronningensgade 68, 3, 1420 Copenhagen, Denmark; 3 Département Biomédical Et Santé Publique, Institut de Recherche en Sciences de La Santé, Ouagadougou 03, BP 7047, Bobo-Dioulasso, Burkina Faso; 4 ALIMA, Route de l’Aéroport, Rue NG 96, BP 15530, Dakar, Sénégal; 5 Childhood Nutrition Research Centre, UCL Great Ormond Street Institute of Child Health, 30 Guilford Street, London, WC1N 1EH UK; 6 London School of Hygiene and Tropical Medicine, Faculty of Epidemiology and Population Health, Keppel Street, London, WC1E 7HT UK; 7 Center for Child Health Research, Tampere University, Faculty of Medicine and Health Technology and Tampere University Hospital, Lääkärinkatu 1, 33014 Tampere, Finland; 8 Department of Paediatrics and Adolenscent Medicine, Blegdamsvej 9, 2100 RighospitaletCopenhagen, Denmark

Correction to: *Scientific Reports* 10.1038/s41598-020-69987-9, published online 06 August 2020.

This Article contains a repeated error in Figure 1 where ‘WHZ’ should read ‘WLZ’.

The correct Figure 1 appears below.

**Figure 1:**
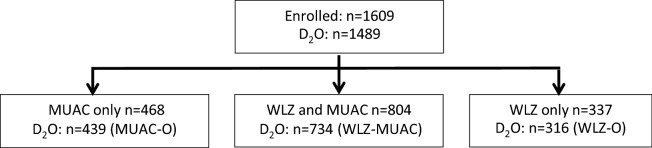
Participant flow chart. Adapted from Ref.^18^. Body composition assessment by deuterium dilution (D_2_O). Mid-upper arm circumference (MUAC). Weight-for-length z-score (WLZ). Children with D_2_O admitted by MUAC only (MUAC-O). Children with D_2_O admitted by WLZ and MUAC (WLZ-MUAC). Children with D_2_O admitted by WLZ only (WLZ-O).

